# “I Am Not Taking Sides as a Female At All”: Co-Facilitation and Gendered Positioning in a Domestic Abuse Perpetrator Program

**DOI:** 10.1177/0306624X241254699

**Published:** 2024-05-30

**Authors:** Helen Cramer, Nathan Eisenstadt, Helena Päivinen, Kate Iwi, Chris Newman, Karen Morgan

**Affiliations:** 1 University of Bristol, UK; 2 University of Jyväskylä, Finland; 3 Respect, London, UK; 4 Partner Abuse Consultancy & Training, London, UK

**Keywords:** intimate partner violence, spouse abuse, gender-based violence, domestic violence perpetrator program, batterer program, group dynamics, behavior therapy

## Abstract

The facilitation of domestic abuse perpetrator programs (DAPPs) by mixed gender co-facilitation pairs brings different facilitator perspectives and enables the modeling of egalitarian and respectful male-female relationships. This study analyzed 22 video and audio recordings of community-based DAPP groups featuring male participants, and male and female facilitators. Using thematic analysis, we aimed to understand how facilitators engaged participants and whether the facilitator’s gender affected this. We found an asymmetry in the positioning of the facilitators. Group participants challenged both facilitators, but especially the female facilitators. Facilitator strategies toward behavior change included softening direct challenges (female facilitators) and mobilizing the shared category of men (male facilitators). Implications from this study are for reflective practice in facilitator management and supervision specifically focused on gendered power dynamics. Skilled facilitation is key to behavior change and the gendered interplay within groups may be a crucial element in the reduction of interpersonal violence and abuse.

## Introduction

What goes on inside behavior change groups for abusive men as part of domestic abuse perpetrator programs (DAPPs) is not well understood. DAPPs are usually by led by mixed gender facilitator pairs and previous research has contended that male and female co-facilitators play different and yet complementary roles (Apps & Gregory, 2011; Roy et al., 2013). Taking advantage of unique access to video and audio data of DAPP groups-in-action, this paper takes a closer look at facilitation strategies-in-progress and gendered group processes: interactions that occurred between male and female co-facilitators, and between facilitators and group participants.

### Gender and Intimate Partner Violence

The unequal gender order and the gendered beliefs, expectations, and behaviors that co-constitute and reinstate that order, is thought to be one of the most powerful causes of intimate partner violence (IPV; Dobash, 2004; Hester, 2013; Pence & Paymar, 1993). Men’s violence to (known) women can be understood as part of a system of power and oppression that constitutes and reinforces patriarchy and patriarchal social relations (Dobash & Dobash, 1979; Hearn, 1998). While anyone can be a perpetrator of IPV, the majority of abusers are men who have relationships with women (Heise et al., 2002) and women are known to experience a higher frequency of incidents, severity of harm, and impacts—both in terms of severity and duration of abuse (Hester, 2013; Walby & Towers, 2018). IPV victimization intersects with other axes of inequality such that poorer, younger, housing-insecure, migrant, Black, Indigenous, and minoritized women, trans and gender non-conforming women, and women have who have experienced IPV as a child are more likely to be affected, and less likely to seek and achieve justice (McCormack & Lantry, 2022). Furthermore, studies have shown that victim/survivors in heterosexual relationships tend to experience those relationships as highly patriarchal and gendered—for example, being positioned as the “home-maker” while her abuser is the “decision-maker” (Women’s Aid et al., 2021). Holma et al. (2006) note a seeming inconsistency in the discursive practices of male perpetrators on their group programs: that violence between men was somehow “honorable” whereas violence from men toward women was not even worthy of mentioning and could be related to masculine identity and the “obligation to protect and the undisputed right to correct or punish” (p. 74).

### Domestic Abuse Perpetrator Programs

Perpetrator programs aim to reduce abuse and increase safety for victims and survivors of IPV. DAPPs often combine group work with safety planning and support for partners and ex-partners, alongside information sharing between multiple agencies to monitor and manage risk (Blacklock, 2003). As the majority of perpetrators are men who have relationships with women, DAPP groups are typically comprised of male participants, although not exclusively (Cannon et al., 2016). Programs vary from highly structured—following a program manual with specific aims and topics to be covered in each session to relatively unstructured, using a set of principles to guide sessions, and taking a more psychotherapeutic approach (e.g., the Finnish “Jyvaskyla model,” see Päivinen & Holma, 2012). In the United Kingdom (UK), DAPPs are typically structured, combining feminist psycho-education, gender role re-socialization, and psychotherapeutic elements (Blacklock, 2003; Nosko & Wallace, 1997; Philips et al., 2013; Roy et al., 2013). The group format (as opposed to one-to-one work) has advantages (Turner et al., 2023) and may help disrupt notions of abuse as an individual phenomenon, especially if links are made with patriarchy and male entitlement (Oba, 2021).

### Previous Research on Domestic Abuse Perpetrator Programs

Despite the widespread and increasing availability of perpetrator programs worldwide the evidence to date remains inconclusive about the degree to which DAPPs work and for whom (Cheng et al., 2021; Nesset et al., 2019; Vigurs et al., 2016; Wilson et al., 2021). The uncertainty about program effectiveness is partly due to challenges in program design, poorly described interventions, poorly measured outcomes, an over-reliance on police incident report data, a lack of victim/survivor data, heterogeneous populations, high attrition rates, and a reliance on short duration of follow-up (Akoensi et al., 2013; Gondolf, 2004, 2012; Lilley-Walker et al., 2018; Turner et al., 2023). Non-randomized evaluations and reviews have reported positive benefits of DAPPs including reduced aggression, abuse, and controlling behavior, the ability to identify and deescalate anger, gaining a more holistic understanding of IPV; gaining a greater sense of accountability for behaviors, improvements in communication skills, conflict management skills, parenting skills, and self-awareness, increased empathy, and emotional regulation (Kelly & Westmarland, 2015; McGinn et al., 2020; Morrison et al., 2018; O’Connor et al., 2021). Reflecting on what counts as success in DAPPs, Westmarland and Kelly (2013) argue that for victim/survivors, success includes increased freedom, the ability to live a full life, to have a respectful relationship, and share positive and safe parenting.

Factors that have been identified as predictive of better outcomes for perpetrators attending programs and their families are: the use of motivational interviewing techniques (Pinto E Silva et al., 2023); a stronger working alliance between facilitators and attendees (Alldredge et al., 2021; Fowler et al., 2021; Santirso et al., 2020); and those with a stake in conformity—perpetrators who are more invested in the values of a society such as those that are married or employed are likely to do better (Mach et al., 2020). As programs usually encompass more than just a group intervention, the wider coordinated community response and increased accountability are considered crucial to success (Gondolf, 2012) as is the integrative partner support work (Chung et al., 2020; McGinn et al., 2021). Factors singled out as barriers to change include: cognitive distortions (such as fixed views about relationships, violence, and the “way of the world”), emotional dysregulation, and low self-esteem (McGinn et al., 2020). Perpetrators of IPV join DAPPs for a range of “push and pull” factors such as being a father, a desire to change or because attendance helps them to avoid custody (McGinn et al., 2020). Perpetrators may be at different points in the stages of change model (precontemplation, contemplation, preparation, action, maintenance, relapse, see Prochaska & DiClemente, 1983) which is also likely to affect their engagement (Fowler et al., 2021).

The group format is central to achieving some of the identified benefits of DAPPs including an opportunity to learn from others, holding each other accountable, feeling less alone and the motivation from seeing other make progress (Morrison et al., 2019). Downsides of the group format comes from disruptive group members, such as dominant speakers and those who are indifferent (Morrison et al., 2019). Using humor and swearing are thought to characterize male DAPPs’ and Hughes (2025) argues that initial resistance and hostility due to shame and anxiety usually develops fairly quickly into a more positive orientation. Holma et al. (2006) note a seeming inconsistency in the discursive practices of male perpetrators on their group programs: that violence between men was somehow “honorable” whereas violence from men toward women could be related to masculine identity and the “obligation to protect and the undisputed right to correct or punish” (p. 74).

### The Theoretical Advantages of Mixed Gender Co-Facilitation

Many DAPPs work to national or regional standards of delivery and duration and it is commonly recommended practice to have both a female and male facilitator leading the group work (Apps & Gregory, 2011; Respect, 2022). Mixed gender co-facilitators can model an egalitarian relationship and, within that relationship, create opportunities to highlight and challenge gender stereotypes. Male and female facilitators are thought to bring different elements and perspectives useful to behavior change work. For example, female facilitators are thought to bring perspectives that are more representative of male perpetrator’s partners and ex-partners (Agustinovich, 2004; Morrison et al., 2019; Päivinen & Holma, 2012; Tyagi, 2006) which may help men to better understand the impact of their abusive behavior (Päivinen & Holma, 2012) increase empathy for victims (Roy et al., 2013) and provide another level of accountability (Apps & Gregory, 2011). Female facilitators may help to defuse fears of intimacy and vulnerability with an all-male group (Tyagi, 2006) and may be more sensitized to recognize certain abusive behaviors that their male colleagues do not perceive, such as subtle gender bias language and entitlement (Agustinovich, 2004; Apps & Gregory, 2011; Blacklock, 2003; McCormack & Lantry, 2022; Roy et al., 2013). Female facilitators may allow male group participants to have immediate and real experiences of engaging with women about their abusive behavior and provide opportunities to learn how to interact with women in a respectful way. Male facilitators may model positive masculinities; that are non-abusive and not complicit in supporting hegemonic masculinities (Connell & Messerschmidt, 2005). Male facilitators may also be able to use some of their own experiences and disclosures to build trust (Roy et al., 2013). Offering experiences to build trust in the same way as their male colleagues is less of an option for female facilitators, as an equivalent offered experience may trigger negative and distancing reactions (Agustinovich, 2004; Blacklock, 2003).

### A Caveat Against Essentialism

We use “male” and “female” to refer to the facilitators perceived gender in the eyes of participants. Thus, a “female” facilitator is one who is perceived to be a woman by participants. This distinction is important because a person’s sex, gender identity, and gender expression (their presentation in relation to gendered norms around clothing, hair, makeup, mannerisms, voice, and interests) is historically and socially constructed (Butler,1990; De Beauvoir, 2015; Martin, 1992). For our purposes here, we are concerned with how a facilitators’ gender is perceived. We use the shorthand “male” and “female” here to refer to this perception rather than making any claim about their sex or gender identity. While there is nothing essentially “male” or “female” about facilitation per se, facilitators being read as “male” or “female” by participants is thought to impact on their engagement in different ways. Thus, a facilitator identifying as a man, and being “read” (understood) as a man by a participant may impact on how a participant responds to him. This response is not due to any inherent property of “being a man,” but rather, the socially trained biases, expectations, and ideas that a participant has about men and how to respond to them. This is important especially in relation to the claims like female facilitators being better able to mobilize empathy for the victim/survivor in work with male perpetrators. If taken uncritically, this claim can imply that there is something inherent in women which positions them as better able to carry out emotional labor—this in turn implies that women should do this work—the essentialist claim thus works to reinforce a social stereotype, and more, one that the group specifically aims to undo. Thus, it is important to make clear that the female facilitator’s observable advantage in making the link to victim empathy is about the participant’s preconceptions rather than being about something essential to her status as a woman.

### Gendered Positioning and repositioning in Group Work

Evaluations by participants of male and female co-facilitators of DAPPs will be influenced by gendered assumptions and stereotypes (Roy et al., 2013) and facilitators may reject the gendered positions they are invited to fulfil by participants and discursively “reposition” themselves (Päivinen & Holma, 2012). Both facilitators are likely to come in for criticism by participants: female facilitators for transgressing traditional and normative gendered social roles by taking on a leadership role (Bernardez, 1983); male facilitators for not retaining a more dominant leadership role which may be considered inadequate and shameful (Agustinovich, 2004; Bernardez, 1983; Blacklock, 2003; Nosko & Wallace, 1997). Male participants are likely to challenge group facilitators about the usefulness of DAPPs, may jockey with each other for status, blame women, refuse to respond to female facilitators, and perceive female facilitators as overly dominating (Bernardez, 1983; Nosko & Wallace, 1997; Päivinen & Holma, 2012). New group norms posed by a DAPP may initially threaten group members’ identity and the DAPP process as a whole may be perceived as an attempt to feminize the men (Hughes, 2025; Morran, 2022). How men interact with the facilitators is part of the process of group work and responses to female facilitators can reveal aspects of men’s attitudes toward women more generally, which can be particularly useful for DAPP group work (Blacklock, 2003; Päivinen & Holma, 2012). Helping men to unpack gender norms and gender expectations is also considered to be an important strategy for reducing abuse, especially coercive control (Downes et al., 2019). Making a case for the examination of gender in DAPPs, Tyagi (2006) argues, “the locus of the work is gender, gender relations and women’s subordination on the basis of gender. Not attending to these processes serves to reinforce the same stereotypes and gender behaviors that this type of counselling tries to address” (p. 17).

### Research Gaps and Questions

Much of the research so far on mixed gender co-facilitation of DAPPs has been drawn from reflections of practitioners (Morran, 2008; Nosko & Wallace, 1997; Oba, 2021; Tyagi, 2006) or from interviews/focus groups with facilitators and perpetrators (Apps & Gregory 2011; Blacklock, 2003; McCormack & Lantry, 2022; Roy et al., 2013). With few exceptions (Hughes, 2025; Päivinen & Holma, 2012; Renehan, 2021), there has been little research based on direct observations of groups and facilitation-in-progress. This study is based on video and audio data from a UK DAPP and captures some of the interactions that occurred between facilitators and participants. The paper builds on the previous body of research and adds detail and substance to some of the challenges that facilitators face. The research questions underpinning this paper are:

RQ1: How does a facilitator’s gender affect the way that they are perceived and responded to in behavior change groups for heterosexual men who have been abusive to their female partners?
RQ2: As role modeling gender equality among mixed-gender co-facilitation pairs is thought to be important in behavior change groups, does this consistently happen?
RQ3: Were there differences in the ways that male and female co-facilitators approached behavior change work with abusive men?

## Methods

This was a qualitative study examining group processes in a DAPP. The study was informed by critical discourse analysis (CDA)—a form of analysis which is particularly interested in social issues (van Dijk, 1993). Discursive meanings are created through social groups which construct and contest shared understandings about the use of language and about what is “normal’ (Gee & Handford, 2012). In analyzing discourses, Meyer (2001) suggests, CDA is often (although not always) perceived as a hermeneutic process. In other words, this is a process of interpretation whereby in order to understand the meaning of a statement, we have to look at the whole world-view of which that statement is part—the hermeneutic circle (Ricouer, 1981).

Critical discourse analysis is a useful reference approach because it not only looks at the content of what is said but to the ways in which speech is made powerful by a social or cultural context, and the power-effects of particular forms of speech. Whilst there is not one specific CDA method—in fact, Van Dijk prefers to refer to Critical Discourse *Studies* (CDS) in order to avoid the misconception that CDA is “a method” rather than a critical perspective (van Dijk, 2008), Fairclough (1993, p. 135) describes CDA as a form of analysis which explores the relationships between discursive practices and social structures. Consequently, it becomes possible to to expose power relationships (Meyer, 2001) and to examine how discourses connect to power. In particular, a feminist approach to CDA interrogates the way in which unequal gendered power relationships and gendered ideologies can be perceived as “common sense,” and, crucially for the focus of this study, how they might be challenged (Lazar, 2017).

As a video analysis based largely on video recordings of groups, following Knoblauch (2012) our approach to video analysis means that: (1) the work involves recordings of social interactions; (2) the work is typically focused on the study of “natural settings” and because of this, there are some clear overlaps with ethnographic approaches; and (3) the work is a qualitative and fundamentally interpretive endeavor. Video analysis is able to register ongoing social activities in very detailed ways that preserves its sequential organization, but also requires simultaneous interpretation (of face formations, gestures, and speech sequences). Video analysis necessarily requires additional contextual knowledge, with Knoblauch and Schnettler (2012) stating that “in order to make sense of the recorded interaction—which is indeed the main object of analysis—the context it is embedded in has to be considered systematically” (p. 335). Video analysis can be used as raw data, in triangulation with other methods (such as interviews), or as a way to help remember and reflect (Toraldo et al., 2018). Video analysis is unique in that it can respond to the problem of “elusive knowledges” providing “a scaffold for translating embodied, tacit and aesthetic knowledge into discursive and textual forms” (Toraldo et al., 2018, p. 438).

This research was embedded within a UK pilot study for a randomized controlled trial of the effectiveness of DAPPs (see also Cramer et al., 2024). To be eligible for the study, male participants had to be aged 21 years or over and be in (or recently had) a relationship with a woman. Participants had to understand enough English to be able to participate in a group program and give informed consent to join a research study. The groups were located in a community setting and attendance on the DAPP was voluntary. The groups occurred weekly for 23 weeks and were delivered by a Respect^1^ accredited IPV organization (https://www.respect.uk.net/pages/accreditation). The groups followed a structured and manualized program with core components, which also allowed for a flexible and responsive approach to issues shared and discussed by participants. The core program components included: safety planning in the early stages; work with men to increase capacity to “straight talk” and explore denial; educational work, for example, to widen men’s definition of abuse; developing a critical awareness of attitudes, beliefs, and expectations that support the use of violence and abuse; building empathy for victim/survivors (e.g., through role play); and identifying and practicing alternative behavior.

The groups were led by a mixed gender co-facilitator pair and there were four facilitators in total. All of the facilitators had a White British background. One of the facilitator pairs had worked together before, the second pair had not. The program manual was new to the facilitators although three out of the four were familiar with some of the content as they had delivered similar material on other programs. The groups typically had between 5 and 8 men attending and there were 11 men featured in the videos. The men ranged in age between 23 and 57 years and out of the 11 men, 7 had a White British background, 3 had a White European background, and 1 had an Asian Indian background. Although the program was not mandated but attended voluntarily, the majority (92%) of men in the pilot study appeared in police records when a search was conducted on the local police force database.^2^ The incident types reported on the police database included: domestic incidents, assault occasioning actual bodily harm, rape, breach of conditions of injunction against harassment, sending letters with intent to cause distress or anxiety, stalking, and threats to kill. The data was collected between August 2017 and August 2018. It is standard practice for UK DAPP group sessions to be videoed, with cameras typically trained on facilitators rather than group participants. We could therefore both see and hear what facilitators were doing, but were usually only able to hear what the men said. The participants all gave written permission for the groups to be observed and analyzed. Ethical approval for the study was given by South Central - Hampshire B Research Ethics Committee (Reference 17/SC/0096).

Out of a possible 31 group sessions recorded, 22 were analyzed. This comprised 15 videoed sessions (approximately 30 hours) and field-notes from seven observed-only sessions. The field-notes provided a supplement to the videos, offering additional visual and other sensory data, and including some facilitator reflections after the groups ended. We selected videos to represent: early program sessions and later sessions when the groups were more established; facilitator pairs in different combinations (e.g., two male facilitators covering sickness, etc.); and sessions likely to be most impactful on participants as identified by facilitators and two public participant involvement (PPI) groups (victims/survivors group and perpetrators group who had attended a DAPP). The sessions considered most impactful were “what is abuse?”, “sexual respect,” and “impacts on children.” The total number of videos and field notes analyzed was related to the constraints of time and funding.

Three researchers (HC, KM, and NE) initially watched and transcribed one video of a group session and from this developed a template for anlaytical purposes. We then viewed and transcribed the other videos, completing a template for each one. These templates captured what was said (verbatim speech where the data was rich and summarized speech when participants veered off topic), observed dynamics, captured researcher interpretation, and emerging themes. We coded for what was said, how it was said and, in keeping with the principles of critical discourse analysis, took into account what we understood as “not said.” Implied (unspoken) meanings can be useful in revealing shared understandings of what may seem to the speaker(s) to be “commonsense” and thus, can be particularly valuable (Fairclough, 1993; Meyers, 1997). This also helps to reveal the connections between what has been said and wider social practice (Fairclough, 1992), which in turn highlights ideological and hegemonic discursive practices which reproduce power relations.

We analyzed the videos for rhetorical strategies, their function, who was speaking for whom, lines of argument being formulated and “positioning.” Positioning refers to a process of social interaction whereby individuals become produced through discursive practices in which they participate. What a person says can position another and a person can also position themselves (Davies & Harré, 1990). Similarities and irregularities across the dataset were considered. From the completed session templates we drew emerging themes together into a coding-tree and then discussed, reviewed and refined these codes with our stakeholder group comprising pratitioners and academics (CN, KI, and HP). Completed templates and group observation notes were uploaded into the qualitative data software package NVivo 12 and coded. Six of the video templates and one observed session were double-coded. The three researchers met regularly to discuss emerging findings, theorize, and shape sucessive iterations of the coding framework. The observations and field notes that we used largely confirmed the findings from the analysis of the videos, and on occasion they sometimes strengthened the findings. For example, after one group a female facilitator directly stated that she sometimes felt under attack from individual participants. Strong themes emerged around different facilitation skills where we felt the gender of the facilitator seemed to matter, either in what they were saying or doing and/or how they were positioned by others. We gave our PPI groups anonymized short extracts of video transcripts to read, discussed possible interpretations of the data and later consulted both the PPI groups on early findings. We gathered feedback and reflections on our emerging findings with the participating facilitators and a wider group of practitioners and managers. In this paper the four facilitators are denoted in the following way: Female facilitator 1 (FF1); female facilitator 2 (FF2); male facilitator 1 (MF1); male facilitator 2 (MF2). Participants are denoted A, B, and C. There were two main groups and pairings: Group A and Group B.

## Findings

The study revealed some of the complexity of group work in DAPPs—discursively fast paced with gender as a constant theme, performed, and problematized in and through the facilitators’ and participants’ speech, behavior, and facilitators’ working relationships. The paper will consider in turn: the asymmetrical positioning of the co-facilitators in the groups by participants; co-facilitator relationships with each other; and co-facilitator strategies for achieving behavior change.

### Asymmetrical Positioning for Male and Female Facilitators

The male and female facilitators were positioned very differently (asymmetrically) in the groups by the program context, content, and by participants. The groups were places where men who were known to be abusive to women were encouraged to openly and verbally reflect on their relationships. What the facilitators faced differed significantly by gender but was also affected by their age, confidence in group work and level of experience in DAPPs. The first example of asymmetrical positioning occurred in the first few weeks of a group’s formation just after a role play exercise where the facilitators played a couple having an argument (see Box 1). The aim of the role play was to show a fairly uncontroversial situation where it was likely to be clear to most participants that the man’s expectations and his behavior toward his partner were unreasonable. However, the aims of role play (acceptance of some principles of equality in relationships) were not reached and, having been aligned with the role of a wife, the female facilitator’s views were dismissed. The facilitator pair was an older male facilitator and a younger female facilitator.

**Box 1.** 6.9.17 Group A.

*As the role play comes to an end. . ..* *A: [laughing along with others in the room] It’s disrespectful and it’s not validating each other and not giving each other acceptance* *B: Is she supposed to be a wife yeh? Well then if you are married to him then you [to FF1] should be getting his gym kit. [B says this in serious tone, FF1 and MF1 still laughing]* *FF1: He should be getting his own gym kit!’* *B: No, that’s the way I see the country now. . .[ ] No offence to you now [B waves his hand towards FF1 then turns to MF1] but it’s a woman’s world now. It used to be a man’s world do you know what I’m trying to say?* *FF1: So you’re telling me. . .?! [laughing, mouth open with mock surprise]* *B: You should be doing the household and doing the cleaning, that’s a woman’s job isn’t it? That’s how I got brought up. . .[ ] I’m an 80s baby. So my family brought me up a different way to the way you’d bring your kids up now. . .[ ]* *[MF1 now has a serious looking face and is listening attentively and patiently]* *FF1: Yes it used to be that women would work less . . ..yes but we are in 2017 now! [FF1 laughs. B and FF1 talking over each other]* *B: Look who’s running the country - a woman . . . [ ] Maybe because it’s the I’ve got two sisters yeh and my mom was always saying. . ..[ ] I can’t deal with you, those girls are so much better than you. . .[ ] I got pushed to one side.*

In this excerpt although two of the three participants seemed to accept and start to verbally agree that the man in the role play was being unreasonable “it’s disrespectful and it’s not validating each other,” one participant rejected the consensus that was beginning to be shared. The participant who objected refers to the legally sanctioned state of marriage as a discursive strategy to justify his position and legitimize men’s claims to women’s services: “if you are married to him then *you* should be getting his gym kit.” Positioned as a representative of all (married) women, the female facilitator starts to respond to the man from an equality perspective. Softening her challenge with a laugh, the female facilitator says “he should be getting his *own* gym kit.” The back of the man’s head is visible in the camera and he dismisses the female facilitator with a hand gesture. The man then turns to the male facilitator saying “it’s a woman’s world now, it used to be a man’s world, do you know what I am trying to say,” possibly in a direct attempt to bond with him. The participant advances his line of argument by co-opting an equality discourse positioning men as victims and referencing the then female UK prime minister (Theresa May) as evidence of increasing and ubiquitous female power. He links his perception of female domination to his own childhood where he felt his mother favored his sisters “my mum was always saying. . ..[ ] I can’t deal with you, those girls are so much better than you.”

As the female facilitator did not agree or confirm the participant’s view of very traditional gender roles where men are entitled to women domestic services, the participant interrupts and dismisses her. Although the female facilitator initially led the responses and discursive challenges, she becomes increasingly silent and, in camera, her body becomes still. Toward the end of the exchange the female facilitator could be seen turning her head away from the participant to look over at the male facilitator, in her body language inviting her colleague’s help to respond to this situation. Like the female facilitator, the male facilitator does get interrupted; but he has also been appealed to. Perhaps deciding that the man’s view were too rigid to be tackled at this point, the male facilitator closed further debate with a brief explanation of equality and invited the female facilitator to re-enact the role play with both parties playing more equal roles.

A second example of asymmetry in the position of male and female facilitators is taken from a mid-program session focused on the impact of abuse where two participants seemed to question the legitimacy of the female facilitator’s right to lead, but not the male facilitator’s. Both female and male facilitators were similar in age and highly experienced in group and DAPP work (see Box 2).

**Box 2.** 14.5.18 Group B.

*At check-in a participant has referred to a recent argument. . .* *FF2: What triggered . . .? [a recent outburst by the participant]* C:[long pause] No, I was asleep . . .. [ ] Literally I just thought what the f***, I’ve made plenty of sacrifices. . .[ ] be honest with me..[ ] if I asked my wife to go and sleep in another room you’d find that odd, do you see what I mean, you know? *FF2: I do* *C: I don’t know if it’s a sexist thing or whatever but it’s the reality of a thing [voice is rising, getting shriller and talking faster]. And yet all I asked was for her to go to another room and she didn’t . . . [ ]* *FF2: Have you spoken to her?* *C: Yeh yeh . . . [ ]* *FF2: Yes, in answer to your previous question then yes, we would have questioned it if you had asked her to leave because you are the one attending the program for previous domestic abuse..[ ]* *C: Would you still think the same if I was not on this program?* *FF2: No, I wouldn’t think the same because I believe in equality.* *D: I think the issue is that a lot of people don’t think like that and I completely see where you are coming from.* *C: Always going to be judged on my past behavior otherwise no point in being here. . .[ ] I think it’s unfair for the person to go ‘No, I am not leaving’. . .[ ] If I had done that, if I had been asked to leave the room and I had said ‘No’, then ‘Oh, that’s abusive!’* *FF2: Why did you ask her to leave the room? Why didn’t you take it upon yourself to leave the room? [Spoken calmly and patiently, legs crossed, head nodding, with one hand holding a mug of tea]* *C:I do every other time . . .[ ]* *FF2: So why was it any different this time?* *C: I knew that I had to get up at 6, at 5 o clock . . . [ ] and I thought that is what Time Out is [taught technique]. . .[ ]* *FF2: No, it’s not how Time Out works generally it’s about how you remove yourself.* *E: I totally empathise with everything you said, I get asked to leave all the time.* *FF2: Yeh I am not taking sides as a female at all.* *E: Yeh but I was going to say unfortunately we are the ones who have perpetrated the abuse so we have got to do a course that we don’t want to do. . . I get reminded all the time [sigh], even when I haven’t actually done anything wrong and it does get . . .* *D: It’s a double standard* *E: Yeh but . . .[ ] you have to think how has it affected the other person. . . [FF2 nodding]. . .[ ]* *MF2: It’s about conversations isn’t it . . .[ ] a conversation about why she feels she needs to do that . . .[ ] but there is a whole battle for you [name] isn’t there. . . [ ]for something that really shouldn’t have got to that point . . .[ ] if you get into a place and then if you think about it the next day thinking ‘I wish I hadn’t done that’ then that’s the bit to change isn’t it?. . .[ ] Thanks [name] for bringing that.*

During the initial check-in with prompting mainly by the female facilitator, a participant had been slowly revealing a recent argument with his partner over her refusal to do something he considered important; sleep in another room when they had been disturbed by a child. The participant asks the facilitator whether his partner’s behavior would ever be considered abusive. Before this question, the man has discursively established himself as a reasonable person by describing his own language toward his wife in largely polite terms and his own actions as being controlled and reflective. He insists several times that on most occasions he is the one to compromise in disagreements “I do every other time” and implies that he thinks the focus on his behavior is unfair “be honest with me” and “if I asked my wife to go and sleep in another room, you’d find that odd” and “I don’t know if it’s a sexist thing?” As the man is out of camera view it is impossible to tell if his questions are directed only toward the female facilitator, but it is the female facilitator who immediately responds by claiming that she does understand, going on to take the “we” position to indicate both her and her male colleague’s position of solidarity. She reminds the man that the program’s core remit is to focus on his behavior not his partner’s. The female facilitator then reinforces her position, defending her right to focus only on his behavior because of his past actions and being on the program rather than because he is a man. Other participants join in supporting the first male participant’s perspective and making comments on a theme of unfairness and inequity, “I totally empathize with everything you said, I get asked to leave all the time” and “it’s a double standard.” While visually looking relaxed and steering the conversation, the female facilitator attempts to de-escalate the emerging gendered divide by eschewing her gender as the reason for her position: “I am not taking sides as a female at all.” A third participant then supports the female facilitator’s right to focus on the men’s actions because of their past abusive behavior. This participant’s reflections however aren’t all useful and, despite his appeal to the other men to take responsibility and recognize the impact of their behavior on their partners, he undermines this when he also implies a superior moral position, self-control, and sacrifice to his partner when he adds “even when I haven’t actually done anything wrong.” The male facilitator wraps up with some measured reflections about power in intimate relationships, partners’ perspectives, and opportunities for change, and through these reflections seems to be supporting his colleague’s position, her initial questions, and her right to be asking questions.

### Lapses in Modeling Egalitarian Relationships

In a stronger status position than their female counterparts in DAPPs (Stahl, 2017), the greater responsibility for demonstrating an egalitarian relationship was with the male facilitators. While a considerable amount of co-facilitator support and respectful communication was in evidence in the data, on occasions there were lapses and one facilitator seemed to dominate or interrupt another facilitator. The first example (see Box 3) is taken from an early session when a co-working relationship was still in development. The facilitator pair was an older male facilitator, skilled and experienced in DAPP group work, and a younger female facilitator totally new to group work and DAPPs. Although the focus here is on gender, it is recognized that it is even harder to model gender equality where a disparity of experience coincides with existing gendered power imbalance.

**Box 3.** 6.9.17 Group A.

*Both facilitators are seated in front of the participants with a stand containing flip chart paper between them.* *MF1: I’ll try and shut up for a bit! [FF1 laughs]* *FF1: What is acceptable and not acceptable behavior in a relationship? [Reads off the manual]* *G: Trying to control* *FF1: Control?* *[MF1 stands up to write on the flip chart, FF2 stays sitting with the manual on her lap]* *H: Fighting* *MF1: Violence? You mean physical? [H nods. MF1 looks over to FF1 for permission to co-lead but is also standing up, holding the pen and inviting the men to speak through direct eye contact]* *G: Belittling* *MF1: [encouraging] Yeh?* *J: Name calling, same sort of thing* *MF1: Yeh.* *J: Using children to get them on your side* G: Yes I find that quite a lot and it p***** me right off. . . [mother of child] says ‘You are not paying attention to your child!’ . . . [ ] I had to walk out the house and disappear for a couple of days. I always find it works, to be fair.

Before the excerpt begins the male facilitator has been leading the session but then explicitly and verbally (“I’ll try and shut up for a bit”) signals that the less experienced female facilitator is going to be leading the next exercise. Observable in the video recording, the female facilitator begins to try and lead the exercise, albeit in an underconfident way by reading directly from the manual in a stilted manner. Although one participant starts to offer a reply and the female facilitator repeats his words (presumably to encourage further elaboration), the male facilitator almost immediately then stands up, takes hold of a pen and physically adopts the role of the interactive note taker. The male facilitator can be seen looking over to the female facilitator as if to acknowledge the leadership is a joint effort. However, he also almost immediately begins inviting the participants to address him rather than his colleague with direct eye contact, verbally encouraging “yeh?” and querying the participant’s responses: “Violence? You mean physical?” The female facilitator soon goes quiet and seems to give up trying to lead. In order to “hold” a group we recognize that a more experienced facilitator will sometimes have to step-in and cover for less experienced colleagues, but in a DAPP context male facilitators and managers have a particular responsibility for addressing this and remaining critically aware how gender imbalances between facilitators may be perceived.

A second example where the egalitarian balance of the co-facilitation relationship seemed to be at risk is taken from a session focused on sexual violence (see Box 4). The facilitator pair was the older experienced male facilitator and younger less experienced female facilitator.

**Box 4.** 17.01.18 Group A.

*MF1 is organising the participants into small groups* *MF1: Have a little bit of a think about um, how we pick up our ideas about our sexual relationships. . .[ ] and I’d like to spend just a few minutes discussing what kind of things we pick up about how sexual relationships are, how they should be, as boys.* *K: What, you want us to talk about our own unrealistic expectations?. . . [ ]* *MF1: Yeah..[ ] this how relationships should be, this is what you are supposed to do as a bloke.* *L: Yeah, but like mine would have been ‘to sleep with as many women as you can’* *MF1: right, [nodding, smiling]* *Group breaks into laughter including both facilitators* *L: Or is that just all blokes?* *MF1:That’s why we want to have a discussion about it because, and we will find, and I’ve actually done this exercise outside the UK as well, in other countries, it’s not that much different.* *L:Yeah, yeah,* *FF1:If you were just on Tinder or Plenty of Fish [dating apps] or something they might just be going over to [inaudible]* *MF1:[interrupting] but those are the details. Our ideas about how we should be as blokes. . . [ ] but I don’t want us to talk about them, I want you to talk about them [MF1 gets up and gestures to the participants to get into groups]* *8 minutes later* *MF1:Shall we come back? Cause we don’t often get to talk about this stuff as blokes do we..[ ] I’d like to hear. . .[ ]* *O: Well we had a really serious conversation about it. We were effectively talking about rape culture – when women cry rape – there’s a couple of instances that have come up in the conversation and how damaging that can be as a man* *P:But it’s happened to me . . .[ ] and now if I go to nightclub I am scared to talk to any girl. . .[ ]* *MF1:So men sometimes have to be careful but women are sometimes at great risk.*

Possibly strategically trying to align himself in the category of men to allow for a better connection and honest discussion to enable relationship building and later challenges, the male facilitator frequently uses the term “we” to emphasize his inclusion in this group “our ideas about how *we* should be as *blokes*” and “we don’t often get to talk about this stuff as *blokes* do *we*?” Although the male facilitator uses the term “we” to refer to him and his cofacilitator, he also takes an “I” position and initially leads the exercise in this way “I’d like to spend just a few minutes discussing.” In this excerpt the male facilitator also draws attention to his expertise running international DAPPs—this, together with the “I” positioning and “how we should be as blokes” discursively combines to position his female colleague as an outsider. When the female facilitator does try to join the conversation with a reference to dating apps, the male facilitator seems to cut her off before she is able to make her point and downplays this potentially useful contribution with “but those are just details.” Although we were not able to ask the facilitators immediately after the group for their views, this dismissal is possibly because the male facilitator was not feeling confident his colleague’s contribution would be helpful at this point. As well as the difficult topic, having been interrupted, positioned as outsider and non-expert, the female facilitator may have felt inhibited and unable to contribute to the discussion, reducing the chance of the gender deconstruction work. Several opportunities to challenge harmful ideas about sexual violence seem to have been missed in this excerpt including the clear mis-understanding of “rape culture” as “a culture of false accusation” which is harmful to men.

### Drawing on Gendered Positions to Facilitate Behavior Change

From the data it was possible to see the facilitators using a variety of strategies to engage the participants, cultivate responsibility-taking for abusive behavior and counter resistance. Some of these strategies either drew on or referenced the facilitators’ gendered position. Although the facilitators risked reproducing traditional gender norms and stereotypes the examples were, in many ways, positive interventions appropriate to the heterosexual relationships of the participants and realistic of the range of possible behavior change in DAPPs (Hearn, 1998). The first example (see Box 5) comes from a session where a participant disclosed to the group that his partner of many years has just ended their relationship.

**Box 5.** 9.5.2018 Group A.

*Q: The work that I have done here, I’m not the volcano that I used to be and that’s let her become stronger, enough to think, ‘You know what, I’m gonna leave ..[ ]* *MF1: And I think that is a factor that does happen sometimes . . . [ ] and a partner might decide I could get out now, it’s safe for me to do so and maybe there’s too much water under the bridge. . .?[ ]* Q: Only last week she was wanting to go away for a romantic weekend in [city]. I just don't get it! I swear. No disrespect [addressed to FF1, in camera view FF1 nods in acknowledgment], but women aren’t f****** wired up the same way that we are. How can you say ‘let's go away for romantic weekend’ and then three days later decide, actually say, ‘that's it! We’re single!’ I don't get it. *MF1: Actually, I do get it. We are all full of contradictory feelings about the same situations. We can feel contradictory things. We can feel really close to somebody but also despairing about the situation. . .. [ ] that swinging backwards and forwards can be quite intense, there’s the . . . [ ] the romantic gesture and the hope of it all. And then there’s the despair of it – ‘I can’t see this ever working’.*

This section starts with the male facilitator encouraging the participant to reflect further on his partner’s perspective and the impact his behavior might have had on her “maybe there’s too much water under the bridge?” The participant then tells the group how just before the split, his partner talked about having a holiday together, something which he says he found illogical and confusing. Discursively positioning himself as a rational man, he moves from talking specifically about his partner to generalizing to all women, a common occurrence in DAPPs (Blacklock, 2003; Päivinen & Holma, 2012). Explicitly positioning the female facilitator in a different membership category: “No disrespect” and “women aren’t f****** wired up the same way we are,” she is then less able to respond in a non-defensive manner. However, capitalizing on his male privilege and insider status, the male facilitator steps into the exchange to suggest an alternative explanation: “Actually I do get it. We are all full of contradictory feelings about the same situations.” The male facilitator here champions and aligns himself with the partners’ perspective. He does not use the term “women” or say, for example, “she may legitimately have contradictory feelings but uses the inclusive gender-neutral terms ‘we all’ and ‘we feel’.” Suggesting a less polarized and united “gender-neutral” approach, the male facilitator further attempts to elicit some compassion and empathy for the mental state of the partner: “swinging backwards and forwards can be quite intense.” It is likely that the male facilitator’s interjection here in defense of an alternative viewpoint made a deeper impact on the speaker and group than if the female facilitator had tried to do the same, having been positioned discursively and symbolically as an outsider just before this exchange.

In the second example (see Box 6) a participant sought emotional support from the female facilitator while smoking outside together in the break. Having been sought out by the participant privately (positioned as both comforter and confidant) the female facilitator disrupts this situation by bringing this (private) conversation out into the (public) space of the group. Although the female facilitator takes the initiative here, she does so in a manner that is unthreatening. The female facilitator seeks agreement for her actions with her male colleague and, as the older and more experienced co-lead in this partnership, the male facilitator uses his privileged position to support and reinforce the legitimacy of his colleague’s interruption. In this way the facilitators worked closely together in complementary ways, albeit aligned with traditional gender norms and roles.

**Box 6.** 12.03.18 Group A.

*R: Yeah, everything’s been fine since [inaudible]* *FF1:So [interrupting but looking at MF1 in support of this interruption] sorry, it’s just that we were talking about it downstairs.* *MF1: Yeah. [Addressed to FF1] Do you want to share anything, or what? [Addressed to participant]* *R:[long pause] Yeah, kind of, best get it out innit?* *MF1:There’s no judgement here [R’s name]* *FF1:[shaking head] No.* *MF1:There’s no judgement here [R’s name], I know it might be difficult, but, it’s good to get it out.* R:Basically, long story short. . .[ ] when I just phoned her. . .[ ] she just ignored me, like reading my messages but. . . didn’t text back, that was absolutely p****** me off but I didn’t say anything. . . [ ] and she finally answered and she was out in town which I don’t mind, again, but when I asked where she was and stuff um, who’s got the kids and she wouldn’t tell me . . .[ ] *FF1:It didn’t go well?* *R:No it didn’t, no it didn’t. . . definitely didn’t. . .* *MF1:Did you end up going down town?*

When asked how he has been, the participant initially reports “everything’s been fine.” The female facilitator almost immediately interrupts and gently challenges the likely continuing discursive direction of this bland statement by encouraging the participant to share something more difficult with the group. This female facilitator softens the directness of her intervention by explicitly apologizing as well as seeming to seek and check her male colleague’s approval beforehand. “So, sorry, it’s just that we were talking about it downstairs.” The male facilitator gives his support and approval for the interruption and then encourages the participant to open up further with “Do you want to share anything” and **“**There’s no judgment here.” Finally agreeing, the participant then shares a description of controlling behavior in his relationship with his ex-partner. This disclosure provided much richer material for the facilitators to work with than would otherwise have been shared in the group, as well as revealing some power and gender dynamics in the group and the facilitator co-working relationship.

## Discussion

Through examination of the interactions that occurred between male and female co-facilitators, and between facilitators and group participants, we have uncovered some key differences for male and female facilitators in group work with abusive men. Benefiting from the unique ability of video analysis to tap into “elusive knowledges” (Toraldo et al., 2018), this paper builds on a body of interview-based research adding detail and substance to some of the challenges that male and female facilitators face. Specifically, how a facilitator’s gender affects the way that they are perceived and responded to, whether role modeling gender equality is maintained and if there were notable differences to the ways male and female facilitators approached behavior change work.

Previous research has contended that male and female co-facilitators play different and yet complementary roles in DAPPs (Apps & Gregory, 2011; Roy et al., 2013). This study confirms the idea of multiple and often complementary roles of facilitators which shifted between positions (adopted, offered and accepted, or resisted) in the groups as they delivered the program material, attended to the dynamics between each other and the group participants, chose what, when and how to try and steer conversations toward greater reflection. However, the positions attributed to male and female facilitators by the male group participants seemed to be qualitatively different. Similar to the findings of other studies, the female facilitator (especially the younger less experienced facilitator) was at times interrupted, dismissed, silenced, isolated, and her words afforded less value, and therefore discursively positioned as relatively powerless, while the male facilitator was more commonly appealed to for a shared understanding (Roy et al., 2013; Tyagi, 2006). Working against normative social roles and so likely to be more negatively evaluated and criticized (Bernardez, 1983; Nosko & Wallace, 1997), at times the leadership role of the female facilitators could be observed to be under threat. Facilitators are meant to represent reasonable and fair authority, and even if female facilitators say exactly the same as their male colleagues, they are more likely to be perceived as biased toward women or the female partners and ex-partners or attacking men (Blacklock 2003; Oba, 2021; Päivinen & Holma, 2012). Their impartial facilitator role is thus liable to challenges around credibility and right to lead the group. Understood through the lens of hegemonic masculinities, in these exchanges we see male dominance and power perceived to be under threat from the perspective of the DAPP participants. In the undermining of the female facilitator, we also see attempts to maintain and re-establish traditional male power (Connell & Messerschmidt, 2005; Hearn, 1998; Hughes, 2025; Morran, 2022; Seymour et al., 2021) and resistance to more fundamental shifts toward deep responsibility taking (Schrock & Padavic, 2007; Seymour et al., 2021). While role plays can help men access concrete material which is less personal and open this up for discussion (Blacklock, 2003), sometimes they fail. Attempts to highlight particular issues such as the unfairness and disadvantages of rigid gender roles may backfire, gender stereotypes are reinforced and female facilitators marginalized (Seymour et al., 2021).

Male facilitators experienced some privilege in the space. The importance of male facilitators being a consistent ally for female facilitators cannot be underestimated (Blacklock, 2003) and male facilitators have a particular responsibility to avoid overshadowing their female colleagues and maintain equality and interchangeability of roles in co-leadership (Nosko & Wallace, 1997). As other studies have noted (Agustinovich, 2004; Apps & Gregory, 2011; Blacklock, 2003; Päivinen & Holma, 2012; Roy et al., 2013; Tyagi 2006), while male facilitators in DAPPS generally sought to mobilize this privilege so as to relinquish or challenge it (and thereby challenge the unequal gender order) they sometimes trod a fine line between cultivating the female facilitator’s own agency and expertise and rescuing, speaking-for, or reasserting dominance in the co-facilitation relationship. Although the co-leadership relationships were observed to have been respectful and egalitarian in the vast majority of cases, we draw attention to the occasional lapses because they are instructive to unpacking and understanding the symbolic and gendered nature of DAPP co-facilitation. Tyagi (2006), argues that unequal co-facilitator relationships are noticed by participants and will impact on the useful work that can be done with the group: “Stereotypical behaviors on the part of the male counsellor only serve to duplicate social relations of power . . . [and] most of [the female facilitator’s] energy is directed at preserving her status, rather than working on the issues being raised” (Tyagi, 2006, p. 15). In many ways it is unfair to critique such complex work in snapshot form and without knowing the facilitators’ longer-term strategies of when best and how deeply to challenge an issue or particular man and when to let something go. However, in these examples such as a discussion of sexual norms, there seemed to be missed opportunities for challenge, to which a more empowered, prepared, and supported female facilitator might have usefully contributed.

Listed amongst Yalom and Leszcz’s (2020) key factors for therapeutic change in group settings are the development of socialization techniques, imitative behavior, and the installation of hope. Role modeling an egalitarian working relationship illustrates what is possible, crucial to re-examining gendered patterns (Oba, 2021). Although men may resist internalizing new masculinities (Hughes, 2025) some argue that accomplishing masculinities differently is needed as men desist from previous abusive behavior (Morran, 2022). If some incorporation of new masculinities is needed, role modeling alternative positive egalitarian masculinities would seem like an important opportunity to support behavior change.

Hegemonic masculinities set many of the “rules” (Connell & Messerschmidt, 2005) within which the facilitators could position themselves and operate. Facilitator strategies toward change included using male privilege and mobilizing the shared category of men to increase connection with the men, reduce reactance, and increase the likelihood of challenges being accepted. Men may have found it easier to accept the advice of a male facilitator, may have felt more understood by him because, as Roy and colleagues report a participant saying, “deep down he’s like us” (2013, p. 15). Asking questions about the impact of men’s behavior on their partners by deconstructing abusive incidents is a way in to increasing empathy (Blacklock, 2003). When male facilitators drew attention to the partner’s perspective and the likely impact of men’s behavior on partners it is likely that their intervention was heard by participants in different ways to when their female colleagues interjected.

Working alliance has been shown to be predictive of behavior change in group therapy (Alldredge et al., 2021) and improves engagement within DAPP groups (Fowler et al, 2021; Soleymani et al., 2018). It is likely these relationships are strongly affected by the facilitators’ gender and their co-facilitation relationship together, and this is worthy of further exploration. An interpretation of what is most skilled, who you trust, and do not trust are all deeply gendered, especially in a group of men who have been recognized to be abusive in intimate partner relationships with women (Blacklock, 2003; Roy et al., 2013).

Where femininity is partly constructed through emotional work within the family (Erickson, 2005), participants may position and single out female facilitators as a source of personal support. Female facilitators may resist such positioning in a variety of different ways but also use the opportunities it affords and, for example, revealing inner vulnerabilities may increase empathy for partners and ex-partners and lead to discussions about the type of men they wanted to be (Oba, 2021). Hughes (2025) acknowledges the discomfort of DAPP participants trying to attain a favorable identity in the groups and noting that there may be differences in their “front and backstage performances.” Again, there are opportunities for facilitators in these differences of identity management. Either consciously or unconsciously, facilitator strategies may align with traditional gender norms, such as a female facilitator checking the acceptability of an interruption with her male colleague in a facilitation style that is less directly challenging.

### Implications for Practice

Female facilitators have been noted as potential “lighting rods” for negative responses in DAPPs that they need to inoculate themselves against (Bernardez, 1983; Oba, 2021; Tyagi 2006). Morran (2008) argues that the emotional impact of dismissive and demeaning attitudes, as well as feelings of shame when they let comments go unchallenged, may result in anger and rage in female facilitators. Furthermore, for female facilitators the wider societal context “of entrenched acceptance of gender inequality and violence against women based on hierarchal structure of power and control” (McCormack & Lantry, 2022 p.6) can feel brutal and if not supported adequately, are unlikely to be confident in their role, at their most effective or be able to sustain longevity of practice (McCormack & Lantry, 2022; Morran, 2008). Practice support and supervision are therefore crucial to address negative feelings that may arise within DAPP work including specific training around how to respond to challenging questions about gender (Renehan, 2021). Supervision needs to be consistent, ongoing, specialized, and comprehensive including clinical supervision and, as needed, mental health support (Apps & Gregory, 2011; Kashkooli-Ellat, 2022; McCormack & Lantry, 2022; Renehan, 2021). Tyagi (2006) lists possible danger signs that managers and supervisors could look out for when considering the impact of group work on female facilitators as well as suggestions for practice. Sociopolitical involvement is suggested as a way for female facilitators to feel less powerless in the face of wider social injustice (Tyagi, 2006). Evans and Hotten (2022) quote a supervisor who reflects on the value of structured regular and documented supervision, especially for addressing equity in co-facilitator relationships: “[it’s]not just about what the men say and how the group was, but about how engagement happened, how co-facilitators went with one another. It encourages those conversations. It encourages thoughtfulness. There’s not this ‘I just go to group and then I leave’. That minimal preparation or that minimal critical analysis about what happened. . .[supervision] explores the power dynamics and co-work dynamics in male and female facilitated groups. It makes sure that equity, or even just strategies around that are a focal point” (Evans & Hotten, 2022, pp. 12–13). Other organizational scaffolding for ensuring facilitator wellbeing is: providing mechanisms for regular feedback and debriefs (Renehan, 2021; Tyagi, 2006); mentoring; and opportunities to join peer support networks (McCormack & Lantry, 2022; Tyagi, 2006). Providing and encouraging uptake of different types of support would also help to mitigate against the feelings of isolation that facilitators are likely to experience due to part time and evening work, and being scattered across sites and regions (Kashkooli-Ellat, 2022).

The importance of male facilitators being a consistent ally for female facilitators cannot be underestimated (Blacklock, 2003; Evans & Hotten, 2022) and male facilitators have a particular responsibility to avoid overshadowing their female colleagues and maintain equality and interchangeability of roles in co-leadership (Nosko & Wallace, 1997). Despite the dangers for female facilitators if their male colleagues do not consistently model gender equality (Tyagi, 2006), facilitator training and support has sometimes been found to be inadequate and insufficient (Kashkooli-Ellat, 2022; Morran, 2022; Renehan, 2021). To support and develop facilitator training, and especially the crucial co-facilitator relationship, we argue that more attention should be focused on gender and power in that relationship. Training of facilitators could encourage less experienced female facilitators to observe more experienced female colleagues and reflections on gender and power dynamics should be encouraged in debriefs. Emphasizing the need for trust building in the co-facilitator relationship, Evans and Hotten (2022) quote a facilitator who says “there’s a lot of this trust building and getting to know each other’s mannerisms and what they need in the space. For the female facilitator, she doesn’t want to be rescued by a man or mansplained though she needs to be able to say that, and when we have the debriefs, we can talk about that really safely” (Evans & Hotten, 2022, p. 12). An example of a gender and power-aware conversation to be encouraged could be: “when I said ‘us guys’ how did you feel? Did you feel supported? If we could re-run that, how would you like it to have gone?” Managers should be specifically seeking conversations about power and gender, and increasing the numbers of female managers could help to achieve this (Apps & Gregory, 2011; Tyagi, 2006). Although different countries may have different practice guidance, facilitator and supervisor training opportunities, delivery standards and methods to assess programs, there are some Europe-wide organizations offering this, such as the Work with Perpetrators European Network (https://www.work-with-perpetrators.eu, see also Tyagi’s (2006) paper as a potentially helpful guide for supporting facilitators). We appreciate that as with some of our study’s examples, modeling equality with large gaps in experience is extremely difficult. However, having adequate time for co-facilitator planning, debriefs and practice support can all minimize and mitigate the imbalances in an unequal relationship and especially when facilitators are new.

Seymour et al. (2021) have outlined a number of ways which men in DAPPs resist change: minimizing, denying, emphasizing only isolated incidents so that little change is needed; emphasizing increased self-awareness and personal development which serves to de-gender and deny the power relations of abuse; and recasting violence as anger which risks overlooking the routinized relations of gender and coercive control. Taken together they argue that men’s resistances undermine the transformative potential of DAPPs and deep responsibility and accountability is lost (see also Schrock & Padavic, 2007). Supporting facilitators to be fully alert to these dangers through robust and ongoing reflections around gender and power is crucial to avoid only superficial engagement of participants and realize the full potential of DAPPS.

### Limitations

Although only snapshots, the strength of this study comes from the detailed unpacking of gendered interactions within DAPPs as they occurred. Whereas previous studies have largely relied on interview data from facilitator and participants, which would be prone to interpretation and recall bias, this study had a unique access to video and audio data of DAPP groups-in-action allowing readers to have interpretations, independent of the authors. Although we did have experienced facilitators on our stakeholder team to help interpret the data, a weakness of the study was not being able to capture and analyze longer trajectories of the facilitators, groups, and individual men and not including the views and reflections of the facilitators who featured in the excerpts enough. It was sometimes difficult to disentangle gender from age and experience and a larger study would be an advantage here. We were also not able to link the instances of facilitation to outcomes such as changes in measures of abuse and the camera was only trained on the facilitators, although arguably a whole room view might have negatively impacted on men’s engagement.

While this small study was based on a DAPP for men who have relationships with women, some DAPPs are not so exclusive and include perpetrators who identify as gay, heterosexual women, transgender, and non-binary people. While gendered positioning of facilitators and participants would no doubt be different in these groups, hegemonic masculinity (Connell & Messerschmidt, 2005) and gender norms are still likely to be relevant. By focusing our analysis mainly on gender we have missed opportunities to explore other structural inequalities in more depth that also impact on the experience and perpetration of domestic abuse (McCormack & Lantry, 2022), such as different group dynamics due to ethnicity, language, age, socioeconomic background, or neurodiversity. Structural inequalities beyond gender need to be addressed and incorporated within a wider examination of group dynamics and power imbalances during debriefs, reflective practice, supervision, and supportive management (Tyagi, 2006). For example, closer attention to the intersectionality of gender, class, and ethnicity may well highlight additional dimensions and dominant power relationships which serve to marginalize specific individuals within the groups (Cooper, 2015).

## Conclusions

Hester and Newman (2020) ask if facilitators in DAPPs are increasingly working in gender neutral ways and abandoning the idea that gender is one of the most powerful distal causes of intimate partner violence. We argue that programs such as the one considered here do place gender at the core of their programs, but that more could be done. Examining gendered expectations and norms may be key in reducing abusive behavior (Downes et al., 2019; Hughes, 2025; McGinn et al., 2020). Unpacking gender norms and assumptions in the group setting requires significant skill: to both support and challenge the men to reflect in ways that they can hear and in utilizing the facilitators’ own gendered positionings. The strength and supportiveness of a co-facilitation relationship is central to successfully managing and holding groups. Managers and supervisors that support the co-facilitation relationship should encourage and deepen facilitator reflective practice, specifically reflective practice around gender in order to realize the transformative potential of DAPPs and avoid reproducing gendered inequalities further.

## Notes

1. Respect is a UK charity and national membership and accreditation body for survivor-focused domestic abuse prevention work.

2. There were 36 men in total in the pilot study. About 33/36 or 92% of the men appeared in the local police force record. The incidents for the total pilot population are given to broadly indicate the criminal justice profile of study participants. The incidents reported are in ascending order from most common (a recording of the police code “domestic incident”) to least common (a recording of police code “threats to kill”).
